# Ethnical Differences in Knee Phenotypes Indicate the Need for a More Individualized Approach in Knee Arthroplasty: A Comparison of 80 Asian Knees with 308 Caucasian Knees

**DOI:** 10.3390/jpm12010121

**Published:** 2022-01-17

**Authors:** Lukas B. Moser, Silvan Hess, Jean-Baptiste de Villeneuve Bargemon, Ahmad Faizan, Sally LiArno, Felix Amsler, Michael T. Hirschmann, Matthieu Ollivier

**Affiliations:** 1 Department of Orthopaedic Surgery and Traumatology, Kantonsspital Baselland (Bruderholz, Liestal, Laufen), 4101 Bruderholz, Switzerland; michael.hirschmann@unibas.ch; 2 Research Group Michael T. Hirschmann, Regenerative Medicine & Biomechanics, Department of Clinical Research, University of Basel, 4001 Basel, Switzerland; silvanhess@msn.com; 3 Aix-Marseille University, ISM, APHM, CNRS, BP 29, 13274 Marseille, France; jbdevilleneuvebargemon@gmail.com (J.-B.d.V.B.); ollivier.mt@gmail.com (M.O.); 4 Department of Orthopedics and Traumatology, Institute for Locomotion, Sainte-Marguerite Hospital, 13009 Marseille, France; 5 Stryker, Mahwah, NJ 07430, USA; ahmad.faizan@stryker.com (A.F.); Sally.LiArno@stryker.com (S.L.); 6 Amsler Consulting, 4059 Basel, Switzerland; felix.amsler@amslerconsulting.ch

**Keywords:** total knee arthroplasty, state-of the-art total knee arthroplasty, Asian, Caucasian, CT, knee phenotypes, coronal alignment

## Abstract

The purpose of this study was to determine the distribution of functional knee phenotypes in a non-osteoarthritic Asian population. The Stryker Orthopaedic Modeling and Analytics (SOMA) database was searched for CT scans of the lower limb meeting the following inclusion criteria: patient age at time of image >18 and <46 years, no signs of fractures and/or previous surgery and/or degenerative changes, Asian ethnicity. A total of 80 CT scans of 40 patients were included (24 males, 16 females). The hip-knee-ankle angle (HKA), femoral mechanical angle (FMA) and tibial mechanical angle (TMA) were measured. Based on these measurements, each limb was phenotyped according to the previously introduced functional knee phenotype concept. All angles and phenotypes of the present study were compared with previously published data of a non-osteoarthritic Caucasian population (308 legs of 160 patients, 102 males, 58 females). Asian knees had a significantly lower TMA (both genders *p* < 0.001) but a higher FMA (males *p* < 0.05, females *p* < 0.001) than Caucasian knees but showed no difference in the HKA. Asian knees differed significantly with regard to femoral and tibial phenotypes (*p* < 0.01), but not with regard to limb phenotypes. The high variability of all coronal alignment parameters highlights the importance of a detailed analysis prior to TKA. Ethnical differences underline the need for a more individualized approach in TKA.

## 1. Introduction

Several studies on total knee arthroplasty (TKA) suggested a connection between a more individualized coronal alignment target and improved clinical outcome [1]. These individualized approaches would require a profound knowledge of the variability of coronal alignment between different patient populations, which unfortunately does not yet exist [2,3]. Whereas gender differences in coronal alignment parameters have been described extensively, ethnical differences remain rather unclear [4,5]. The few existing studies were mainly focused on anthropometric parameters such as medial–lateral and/ or anterior–posterior ratios and showed significant ethnical differences [6,7,8]. Consequently, ethnical differences in coronal alignment parameters would seem natural. However, studies investigating ethnical differences in coronal alignment parameters are rare and their results inconclusive. Some studies reported a more valgus-aligned distal femur (FMA) in Asian knees compared to Caucasian knees [9,10], while others did not find any significant difference [11]. A similar picture evolves for alignment of the tibial joint line (TMA) and the overall limb alignment (HKA).

Recently, Hirschmann et al. introduced the functional knee phenotype concept, which is based on the alignment of non-osteoarthritic knees [12]. This concept allows a comprehensive and collective analysis of all coronal lower limb parameters [13,14] and reveals overall differences as well as femoral and tibial alignment dissimilarities between genders. However, this concept has only been applied to Caucasian knees and has not yet been applied to evaluate ethnical differences in coronal alignment.

The purpose of this study was therefore (1) to apply the functional knee phenotype concept to a non-osteoarthritic Asian population, and (2) to compare the distribution of this population to an already published distribution of a non-osteoarthritic Caucasian population. It was hypothesized that Asian and Caucasian knees do not differ regarding their coronal alignment.

## 2. Materials and Methods

**Figure 1:**
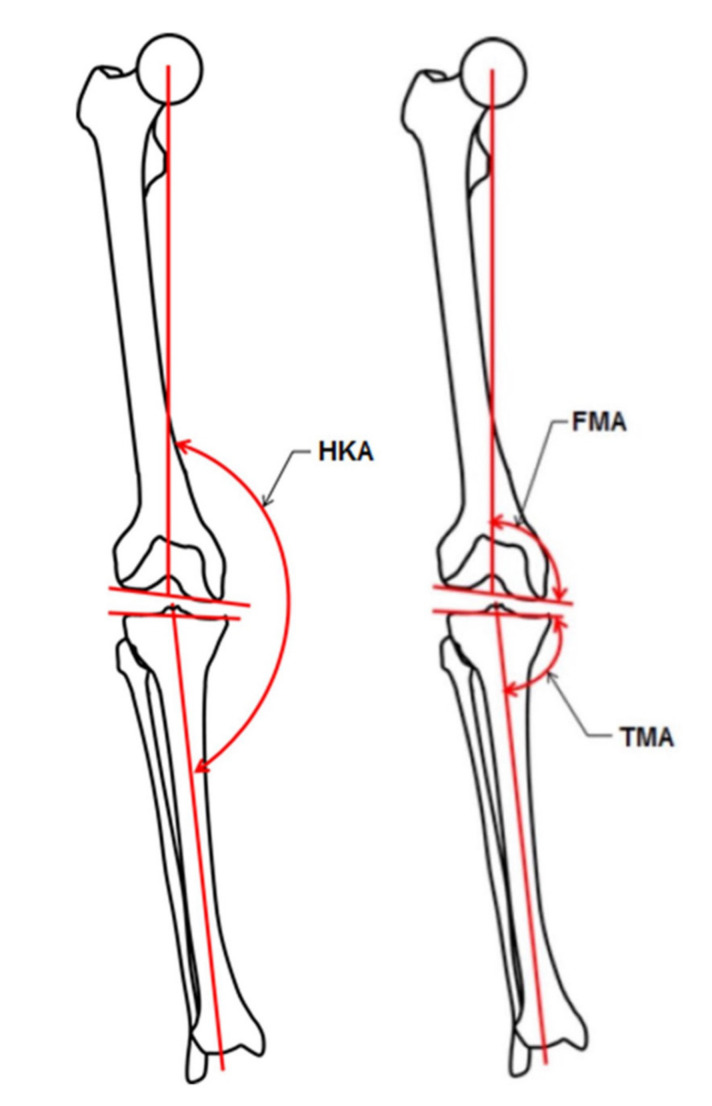
This figure illustrates the measured angles: hip–knee–ankle angle (HKA, the angle is formed by the lines connecting the centers of the femoral head, the knee and the talus), femoral mechanical angle (FMA, the angle between the femoral mechanical axis and a tangent to the distal femoral condyles), tibial mechanical angle (TMA, the angle between the tibial mechanical axis and a tangent to the proximal tibia joint surface).

The SOMA database (Stryker Orthopaedic Modeling and Analytics database, Stryker, Mahwah, NJ, USA) was searched for CT scans of the lower limb meeting the following inclusion criteria: patient age at time of image >18 and <46 years, no signs of fractures and/or evidence of previous surgery and/or degenerative changes (osteoarthritis), patient of Asian ethnicity. SOMA is a database containing over 3500 CT scans that have been segmented to provide 3D models of more than 15,000 individual bones. Whilst the patients whose scans are included in the database are anonymized, basic demographic information (age, gender and ethnicity) is recorded for each. Scans were performed in several institutions around the world and were only eligible for inclusion if the slices were less than 1.5 mm thick and without motion artefacts. A total of 80 CT scans of 40 patients were included (24 males, 16 females). Mean age ± standard deviation (SD) was 34 ± 7 years in males and 33 ± 7 in females. The following angles were measured and reported as mean ± SD and range (Figure 1): hip–knee–ankle angle (HKA, the angle is formed by the lines connecting the centers of the femoral head, the knee and the talus), femoral mechanical angle (FMA, the angle between the femoral mechanical axis and a tangent to the distal femoral condyles), tibial mechanical angle (TMA, the angle between the tibial mechanical axis and a tangent to the proximal tibial joint surface). For the sake of consistency, the medial angle was always reported. Thus, an angle for FMA and TMA of less than 90° means varus, and an angle of more than 90° means valgus.

All measurements were conducted using algorithm-calculated constructions on a reference bone, which were then mapped to each chosen bone from the database. This resulted in reproducible and consistent constructs for each specimen [15,16,17,18]. Previous accuracy and reproducibility analysis estimated that this system allows automated measurement of anatomical parameters in the proximal femur with a margin of error of less than 2 mm or 1° [19].

Based on these measurements, each limb was phenotyped according to the previously introduced functional knee phenotype concept [12]. Table 1 shows the definition of these phenotypes.

The angles (HKA, FMA and TMA) and distribution among the phenotypes of the present study were compared to Hirschmann et al.’s previously published angles and distribution of a non-osteoarthritic Caucasian population [12,13,14]. This retrospective study included 308 knees of 160 non-osteoarthritic patients who received a CT in accordance with the Imperial Knee Protocol. The male-to-female patient ratio was 102:58, and the mean age ± standard deviation was 30 ± 7 years (16–44). The same coronal alignment parameters as seen in the present study were measured by one observer using a previously validated, commercially used planning software that creates 3D models based on CT scans (KneePLAN 3D, Symbios, Yverdon les Bains, Switzerland). For CE marking, the accuracy of measurements including inter- and intra-observer reliability has been reported as excellent, with measurement variability within 1°. 

A professional and experienced statistician F.A (FA) performed all calculations using IBM SPSS Statistics for Windows, version 26.0 (IBM Corp: Armonk, NY, USA). Descriptive statistics such as means, standard deviations, medians, ranges and measures of variance are presented. For independent samples, *t*-tests were used to compare group differences as all angles were normally distributed. In some patients, both limbs were included in the analysis, and thus the relevant N for *t*-tests was set to the number of patients instead of number of limbs. Beckers et al. applied the phenotype system when investigating the left-to-right symmetry of 250 non-osteoarthritic knees [20]. Symmetrical distribution was found in 79% of the overall limb phenotype (HKA), and in only 59% of the limb phenotype (FMA, TMA). This asymmetry supports the strategy to phenotype each limb separately as performed in the present study. Phenotypes were compared with linear-by-linear association tests for ordinal categories. Due to significantly different angles of male and female legs and different proportions of male and female legs in the two groups, values were reported separately for each sex. The statistical level of significance was two-sided *p* < 0.05.

## 3. Results

### 3.1. Basic Alignment Parameters

Table 2 shows the mean values ± SDs and the ranges found in the female and male populations. Females showed significantly higher HKA and FMA values than males. Asian knees had a significantly lower TMA (both genders *p* < 0.001) but a higher FMA (males *p* < 0.05, females *p* < 0.001).

### 3.2. Phenotypes

**Figure 2:**
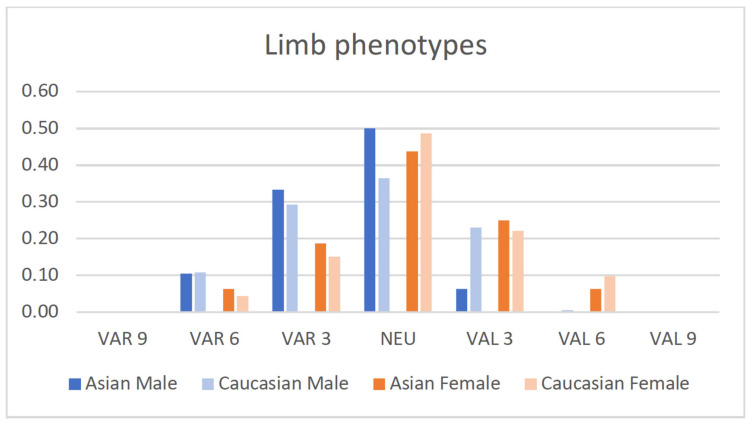
Proportion of male and female limb phenotypes of the present Asian population compared to a previously published Caucasian population [13].

**Figure 3:**
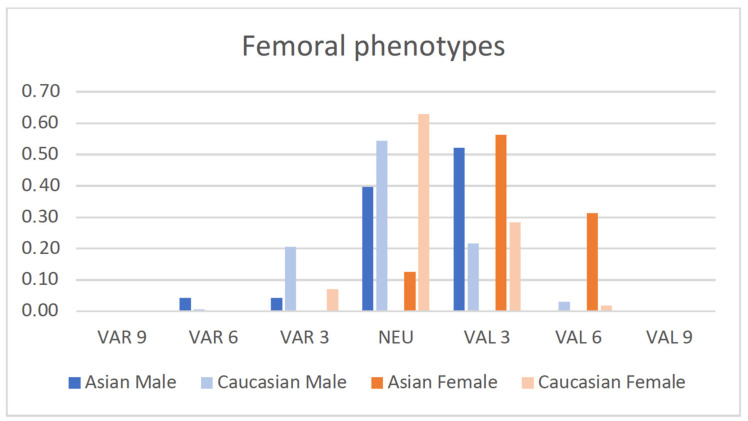
Proportion of male and female femoral phenotypes of the present Asian population compared to a previously published Caucasian population [14].

**Figure 4:**
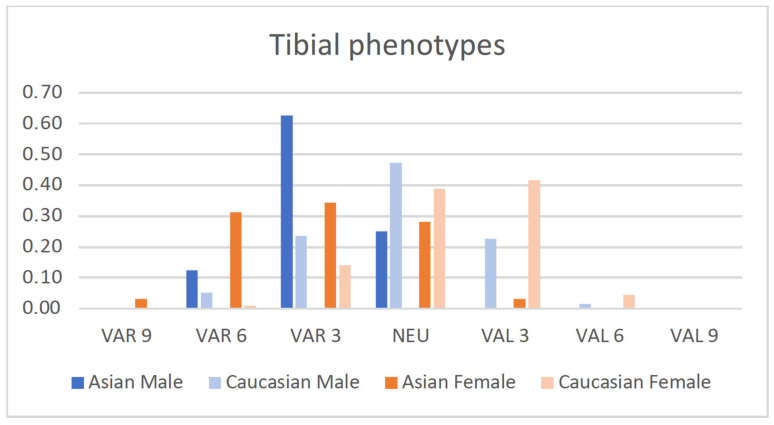
Proportion of male and female tibial phenotypes of the present Asian population compared to a previously published Caucasian population [14].

The distribution of the male and female populations among limb, femoral and tibial phenotypes is shown in Figure 2, Figure 3 and Figure 4.

Asian and Caucasian knees differed significantly with regard to their femoral phenotypes (*p*_females_ < 0.001, *p*_males_ < 0.01) and tibial phenotypes (*p* < 0.001), but not with regard to their limb phenotypes.

Femoral phenotypes confirmed a more valgus-aligned femoral joint line in Asian compared to Caucasian knees: 52% of the male Asian knees had the phenotype VAL_FMA_3°, which was only present in 22% of the male Caucasian knees. This percentage was similar in female knees (56% vs. 28% in VAL_FMA_3°). VAL_FMA_6° was only present in 2% of the female Caucasian population, but in 31% of the female Asian knees.

The distribution of the tibial phenotypes reflects a more varus-aligned tibial joint line in Asian knees compared to Caucasian knees: VAR_TMA_3° was present in 63% of the male Asian knees and only in 24% of the male Caucasian knees. VAR_TMA_6° was only found in 1% of the female Caucasian knees, but in 31% of the female Asian knees.

Combining the femoral and tibial phenotypes of the patients with the knee phenotypes showed that VAL_FMA_3° + VAR_TMA_3° was most common (Table 3). This knee phenotype was found in 29% of the male and 22% of the female Asian knees, but in less than 10% of the Caucasian knees. In male Caucasian knees, the most common knee phenotype was NEU_FMA_3° + NEU_TMA_3° (26%), which was only seen in 6% of the male Asian knees. In female Caucasian knees, the most common knee phenotype was NEU_FMA_3° + VAL_TMA_3° (28%), which was only seen in 3% of the female Asian knees.

The most common functional knee phenotype in Asian knees was NEU_HKA_0° + VAL_FMA_3° + VAR_TMA_3° in both genders (male: 19%, and female: 25%), but this only accounted for less than 3% each of the male and female Caucasian knees. In Caucasian knees, the most common functional knee phenotype was NEU_HKA_0° + NEU_FMA_3° + NEU_TMA_3° in both genders (male: 19%, and female: 18%), but this only accounted for 4% of the male Asian knees, while it accounted for none of the female Asian knees.

## 4. Discussion

The most important findings of the present study are the following:

First, the study hypothesis was rejected as the tibial and femoral coronal joint line orientation of Asian knees significantly differed from that of Caucasian knees. Male and female Asian knees had a more varus-aligned tibial joint line (2.6° and 3.8°) and a more valgus-aligned femoral joint line (1° and 2.8°) than their Caucasian counterparts.

The increased valgus alignment of the distal femur in Asian knees was first described by Harvey et al. (FMA = 96°) using short leg films [9]. Zeng et al. confirmed these findings using 3D-reconstructed CT scans, although the valgus alignment was lower than in the present study (FMA = 93.8° in males, and 94.4° in females) [10]. On the contrary, the increase in the varus alignment of the tibial joint line in Asian knees is less supported in the literature, with just one study using weight-bearing radiographs. Tang et al. investigated the TMA in 50 healthy volunteers from South China and found an increased varus alignment (TMA in males 85.1°, TMA in females 84.6°) compared to studies on Caucasian knees [21].

Secondly, ethnical differences between non-osteoarthritic Asians and Caucasians were not observed for overall limb alignment. The HKA was slightly in varus in male knees and slightly in valgus in female knees in both ethnicities. These findings are in line with previously published findings using non-weight-bearing imaging modalities [13,22]. Nevertheless, comparing the overall limb alignment of weight-bearing imaging (LLR) with non-weight-bearing imaging (CT/MRI) is difficult, as the effect of laxity, for example, might increase the HKA in a weight-bearing position. However, the FMA and TMA rely solely on bony landmarks of one bone, and therefore neither parameter should be influenced by weight bearing. Nevertheless, rotation and flexion of the knee make 2D imaging modalities less reliable than 3D evaluation [23,24]. Reviewing the current literature revealed a lack of knowledge of the coronal alignment of non-osteoarthritic Asian knees in 3D, as most studies used radiographs [9,11,21,25,26,27,28,29,30] (*n* = 9), one study used MRI [22] and only one study reconstructed CT scans in 3D [10].

Our results clearly support findings of recent studies that show classifying the coronal knee alignment only by the HKA in varus, valgus or neutral is short sighted [12]. Assessing only HKA would be misleading, as no difference in HKA was found, although there are coronal alignment differences between Asian and Caucasian knees.

The application of the “functional knee phenotype” concept thereby better represents the individual coronal knee alignment. Phenotyping the knee allows a different perspective on the individual knee anatomy and enables a more profound discussion about ethnical differences [12]. The results of the present study not only reveal that Asian knees have a highly variable joint line orientation of the femur and tibia, but also that their combination (knee phenotypes) in relation to the overall alignment is unique and different compared to Caucasian knees. Asians and Caucasians with the same HKA have different tibial and femoral joint line orientations regardless of overall alignment (varus/valgus/neutral).

Ethnical differences of the femoral and tibial phenotypes might play an important role for the knee surgeon planning for TKA. Mechanical alignment aims to place the femoral and tibial components perpendicular to their mechanical axis [31]. For a long time, it was accepted that the distal femoral joint line is in 3° valgus and the tibial joint line is in 3° varus [32]. Although this might approximately be the case for the average population, severe individual deviations have been reported for Caucasian knees [14]. As a consequence, aiming for a mechanical alignment in TKA results in severe changes in collateral ligament strains and the envelope of laxity in many knees [33]. The present study shows that non-osteoarthritic Asian knees have an increased deviation from neutral in the femur and the tibia (FMA and TMA = 90°) compared to Caucasian knees. Accordingly, mechanical realignment changes the coronal alignment in Asian knees more strongly. Similarly, the concept of anatomical alignment has been developed on the basis of Caucasian knees, and its applicability for Asian knees is questionable as well [34]. The aim of the anatomical realignment strategy is to align the lower limb neutrally (HKA = 180° ± 1.5°) and to anatomically restore the 3° valgus in the femoral joint line (FMA of 87°) and the 3° varus in the tibial joint line (TMA of 87°). Likewise, the present study demonstrates that the native joint line orientation is different in Asian knees. Consequently, despite aiming to restore the natural joint line orientation, anatomical alignment would significantly change the anatomy in most Asian knees.

Whereas mechanical alignment and anatomical alignment follow a systematic, more dogmatic approach, the recently introduced kinematic alignment aims for an individualized alignment strategy [35]. The postoperative alignment of patients treated using this concept mimics the native coronal knee alignment of the individual knee because the pre-arthritic femoral and tibial distal joint lines are restored [36]. This alignment strategy considers the individual anatomy of a patient and thereby offers an approach to diminish ethnical-related differences in coronal alignment [37].

In summary, the high variability of all coronal alignment parameters and ethnical differences in FMA and TMA highlights the importance of a detailed coronal alignment analysis prior to TKA. The functional knee phenotype concept should be applied in order to obtain a more comprehensive understanding of the individual coronal knee alignment.

The present study has a considerable number of limitations: First, the data were derived from a database (SOMA) with only limited patient information. Although we know that most of the patients come from China, Singapore or Taiwan, the exact origin of each patient is unknown. Previous studies suggested differences in alignment parameters within the Asian ethnicity (e.g., between Indian and Japanese patients) which could have biased our analysis [22,27]. Furthermore, the effect of the patient’s height on coronal alignment was not investigated, as height data were not provided by the database. It would have been interesting to compare the alignment parameters to the same ranges of height in both populations. The study on Caucasian knees published by Hirschmann et al. likewise did not provide any data on patients’ height. However, one study by Nayak et al. suggested patient height had no effect on coronal knee alignment. They investigated 966 limbs of 493 Indian patients (56.05% females, and 42.96% males) on long-leg radiographs and did not find any significant association of patient height with the alignment of the limb (*p* < 0.05) [38]. The sample size was small (48 male knees and 32 female knees). Phenotyping 40 male knees revealed 17 different functional knee phenotypes, and phenotyping 32 female knees revealed 15 different functional knee phenotypes. Hirschmann et al. found 35 different functional knee phenotypes in 195 males and 26 different functional knee phenotypes in 113 females. This suggests that the number of different functional knee phenotypes would increase when investigating a larger population. However, this is currently the largest dataset of 3D-reconstructed CT scans in a non-osteoarthritic Asian population investigating coronal knee alignment using the phenotype concept.

## 5. Conclusions

The high variability of all coronal alignment parameters highlights the importance of a detailed analysis prior to TKA. Ethnical differences underline the need for a more individualized approach in TKA.

## Figures and Tables

**Table 1:** Definitions for all phenotypes: limb phenotype (based on hip–knee–ankle angle (HKA)), femoral phenotype (based on femoral mechanical angle (FMA)) and tibial phenotype (based on tibial mechanical angle (TMA)).

Coronal Alignment				Average Value
HKA		VAR_HKA_9°	169.5° < HKA < 172.5°	171°
	VAR_HKA_	VAR_HKA_6°	172.5° < HKA < 175.5°	174°
		VAR_HKA_3°	175.5° < HKA < 178.5°	177°
	NEU_HKA_	NEU_HKA_0°	178.5° < HKA < 181.5°	180°
	VAL_HKA_	VAL_HKA_3°	181.5° < HKA < 184.5°	183°
		VAL_HKA_6°	184.5° < HKA < 187.5°	186°
		VAL_HKA_9°	187.5° < HKA < 190.5°	189°
FMA	VAR_FMA_	VAR_FMA_6°	85.5° < FMA < 88.5°	87°
		VAR_FMA_3°	88.5° < FMA < 91.5°	90°
	NEU_FMA_	NEU_FMA_0°	91.5° < FMA < 94.5°	93°
	VAL_FMA_	VAL_FMA_3°	94.5° < FMA < 97.5°	96°
		VAL_FMA_6°	97.5° < FMA < 100.5°	99°
TMA	VAR_TMA_	VAR_TMA_6°	79.5° < TMA < 82.5°	81°
		VAR_TMA_3°	82.5° < TMA < 85.5°	84°
	NEU_TMA_	NEU_TMA_0°	85.5° < TMA < 88.5°	87°
	VAL_TMA_	VAL_TMA_3°	88.5° < TMA < 91.5°	90°
		VAL_TMA_6°	91.5° < TMA < 94.5°	93°

The first part (NEU = neutral, VAR = varus, VAL = valgus) defines the deviation direction of alignment. The second subscripted part (e.g., HKA) states the measured angle. The last part (0°, 3° and 6°) shows the mean value of the phenotypes, which represents the mean deviation of the phenotype from the overall mean value.

**Table 2:** Mean HKA, FMA and TMA values with standard deviations (SDs) and ranges for female and male knees.

		Asian			Caucasian ^1^			*p*-Value *
	Angle	N	Mean ± SD	Range	N	Mean ± SD	Range	
Male	HKA	48/24	178.5° ± 2.0°	173.7–182.9°	195/102	179.2° ± 2.8°	172.6–184.9°	0.15
	TMA		84.1° ± 1.4°	81.1–86.7°		86.7° ± 2.3°	81.3–94.6°	<0.001
	FMA		94.1° ± 2.0°	87.5–97.4°		93.1° ± 2.1°	87.9–100.0°	0.03
Female	HKA	32/16	180.5° ± 2.8°	174–185.3°	113/58	180.5° ± 2.7°	172.9–187.1°	0.99
	TMA		84.2° ± 2.7°	77.5–89°		88.0° ± 2.3°	82.3–94.0°	<0.001
	FMA		96.6° ± 1.6°	93.7–99.8°		93.8° ± 1.8°	90.1–98.1°	<0.001
Male vs. female (*p*-value)	HKA	0.02			<0.007			
	TMA	0.90			0.001			
	FMA	<0.001			0.03			

^1^ Previously published values [2,13]. * *p*-values were calculated on the basis of number of patients.

**Table 3:** The distribution of female (above) and male (below) populations among the different knee phenotypes of the Asian group.

Females	Femoral Phenotypes	VAR 9	VAR 6	VAR 3	NEU	VAL 3	VAL 6	VAL 9
**Tibial** **phenotypes**	Ranges	82.5°–85.5°	85.5–88.5°	88.5–91.5°	91.5–94.5°	94.5–97.5°	97.5–100.5°	100.5–102.5°
**VAR 9**	79.5–82.5°					1 (3%)		
**VAR 6**	82.5–85.5°				2 (6%)	6 (19%)	2 (6%)	
**VAR 3**	85.5–88.5°					7 (22%)	4 (13%)	
**NEU**	88.5–91.5°				1 (3%)	4 (13%)	4 (13%)	
**VAL 3**	91.5–94.5°				1 (3%)			
**VAL 6**	94.5–97.5°							
**VAL 9**	97.5–100.5°							
**Males**	**Femoral Phenotypes**	**VAR 9**	**VAR 6**	**VAR 3**	**NEU**	**VAL 3**	**VAL 6**	**VAL 9**
**Tibial** **phenotypes**	Ranges	82.5–85.5°	85.5–88.5°	88.5–91.5°	91.5–94.5°	94.5–97.5°	97.5–100.5°	100.5–102.5°
**VAR 9**	79.5–82.5°							
**VAR 6**	82.5–85.5°				3 (6%)	3 (6%)		
**VAR 3**	85.5–88.5°		2 (4%)	1 (2%)	13 (27%)	14 (29%)		
**NEU**	88.5–91.5°			1 (2%)	3 (6%)	8 (17%)		
**VAL 3**	91.5–94.5°							
**VAL 6**	94.5–97.5°							
**VAL 9**	97.5–100.5°							

## Data Availability

The data is not publicly available in accordance with the approval of the Institutional Review Board.
